# Speciation of inorganic arsenic in aqueous samples using a novel hydride generation microfluidic paper-based analytical device (µPAD)

**DOI:** 10.1007/s00604-022-05339-w

**Published:** 2022-06-03

**Authors:** Mason E. Bonacci, M. Inês G. S. Almeida, Yanlin Zhang, Spas D. Kolev

**Affiliations:** School of Chemistry, The University of Melbourne, Parkville, VIC 3010 Australia

**Keywords:** Microfluid paper-based analytical device (µPAD), Arsenic, Speciation, Hydride generation, Flatbed scanner, RGB values, Reflectometry

## Abstract

The development of the first microfluidic paper-based analytical device (µPAD) for the speciation of inorganic arsenic in environmental aqueous samples as arsenite (As(III)) and arsenate (As(V)) which implements hydride generation on a paper platform is described. The newly developed µPAD has a 3D configuration and uses Au(III) chloride as the detection reagent. Sodium borohydride is used to generate arsine in the device’s sample zone by reducing As(III) in the presence of hydrochloric acid or both As(III) and As(V) (total inorganic As) in the presence of sulfuric acid. Arsine then diffuses across a hydrophobic porous polytetrafluoroethylene membrane into the device’s detection zone where it reduces Au(III) to Au nanoparticles. This results in a color change which can be related to the concentration of As(III) or total inorganic As (i.e., As(III) and As(V)) concentration. Under optimal conditions, the µPAD is characterized by a limit of detection of 0.43 mg L^−1^ for total inorganic As (As(III) + As(V)) and 0.41 mg L^−1^ for As(III) and a linear calibration range in both cases of 1.2–8.0 mg As L^−1^. The newly developed µPAD-based method was validated by applying it to groundwater and freshwater samples and comparing the results with those obtained by conventional atomic spectrometric techniques.

**Graphical abstract:**

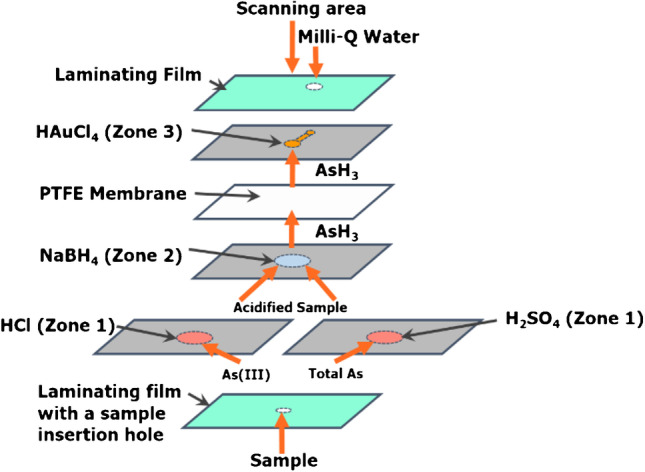

**Supplementary Information:**

The online version contains supplementary material available at 10.1007/s00604-022-05339-w.

## Introduction

Arsenic pollution is of considerable environmental and health concern due to its ubiquitous nature. Inorganic arsenic species, present predominantly as arsenite (As(III)) and arsenate (As(V)), are considerably more toxic than organoarsenic compounds, with As(III) being more toxic than As(V) [1]. Due to the risks arsenic compounds pose to human health, the World Health Organization (WHO) has set the maximum acceptable concentration of arsenic in drinking water to 10 µg L^−1^ [2].

There is also a growing concern regarding heightened arsenic concentrations in waters used for irrigation of crops which could result in subsequent bioaccumulation in plants, particularly rice [3]. For these reasons, the maximum allowed arsenic concentration for short-term irrigation (less than 20 years) as specified by the Australian Water Quality Guidelines for Fresh and Marine Waters is 2.0 mg L^−1^ [4]. However, this guideline value does not account for the fact that the As bioaccumulation in plants is directly affected by the arsenic species present [5]. For these reasons, it is important to have analytical methods that are sensitive and can accurately differentiate between arsenic species and in particular the more toxic As(III) and As(V) species.

A variety of analytical techniques have been developed for the determination of these inorganic arsenic species, including hydride generation atomic spectrometry [6], electrochemical methods based on stripping analysis or amperometry [7, 8], and hyphenated techniques based on coupling chromatographic separation with optical emission spectrometric detection [9]. However, there is a lack of easy to use, inexpensive, and portable analytical tools for on-site screening of environmental waters, including those used for irrigation, for As(III) and As(V).

The determination of inorganic arsenic has been successfully conducted in flow-based analyzers where the determination of inorganic arsenic involves hydride generation of arsine gas from the As(III) and As(V) species in the analyzer’s donor stream with subsequent arsine measurement in its acceptor stream by spectrophotometric [10], chemiluminescence [11], or amperometric [7] detection. The pH dependence of arsine generation can be exploited to perform inorganic As speciation. This approach has been utilized previously by us to develop a sequential injection analysis system for on-line As speciation [12]. Additionally, a number of detection methods have been used for measuring either As(III) or total inorganic As (As(III) + As(V)). These methods include fluorescence, surface-enhanced Raman spectroscopy, surface plasmon resonance, and inductively coupled plasma-mass spectrometry, as well as electrochemical methods [13]. While these flow methods allow automatization of analysis, thus making the corresponding devices user-friendly, many of them utilize instruments with limited portability and this can create difficulties in conducting on-site analysis.

Most commercially available field test kits are based on the Gutzeit reaction, whereby arsine gas is produced via hydride generation. However, they are relatively expensive, often use toxic mercuric bromide, and can potentially expose operators to relatively large volumes of the toxic arsine gas, and their reliability has been questioned [14]. In addition, these test kits are not suitable for inorganic arsenic speciation.

Recently, Thepmanee et al. [15] and Pena-Pereira et al. [16] proposed devices for measuring total arsenic which combine paper-based sensing with conventional-sized reaction chambers where arsine is generated. These systems require a relatively large sample and reagent volumes (in the mL range), which is likely to increase the cost of analysis and could potentially raise some health and safety concerns because of the use of relatively large quantities of hazardous chemicals. In the latter case, i.e., [16], a magnetic stirrer is also used for mixing the reaction mixture in the reaction chamber which reduces the portability of the device.

Whitesides et al. [17] introduced more than a decade ago the so-called microfluidic paper-based analytical devices (μPADs), which have provided the possibility for conducting inexpensive and rapid on-site environmental analysis [18].

Previously developed µPADs have been successfully used for chemical speciation in water samples [19]. The range of analysis that can be conducted by µPADs has been considerably extended in recent years. Jayawardane et al. [20] have demonstrated that the separation of analytes from the sample matrix is achievable on a paper-based platform by designing a µPAD for the determination of ammonia in aqueous solutions based on a gas-diffusion approach. Ammonia gas generated in the device’s sample zone diffuses across a polytetrafluoroethylene (PTFE) tape into the device’s detection zone, thus eliminating possible interferences from chemical species present in the sample matrix.

Existing µPADs for the determination of inorganic arsenic rely on the use of functionalized gold nanoparticles sensitive to As(III) [21–23]. Therefore, these devices have not been used for the speciation of As(V) and As(III). It should also be noted that the synthesis of the functionalized gold nanoparticles is generally complex and time consuming.

For the reasons mentioned above, there is a clear need for the development of user-friendly, inexpensive, and portable analytical tools for on-site inorganic arsenic speciation and the present article reports on the development of such a µPAD and its successful application to the analysis of groundwater and freshwater samples. The newly developed device couples hydride generation under controlled pH conditions for the selective reduction of either As(III) or both As(III) and As(V) with gas-diffusion separation of arsine from the sample matrix. The detection is based on the color change of the rehydrated hydrophilic detection zone containing gold chloride (HAuCl_4_).

## Materials and methods

### Reagents and solutions

All chemicals were of analytical reagent grade, and deionized water (≥ 18.2 MΩ cm, Millipore, France) was used in the preparation of all solutions. Stock solutions (1000 mg L^−1^) of As(III) and As(V) were prepared by dissolving 0.1720 g of NaAsO_2_ (Sigma-Aldrich, Australia) and 0.4182 g Na_2_HAsO_4_ (Sigma-Aldrich, Australia), respectively, in 1 mL of 5.0 mol L^−1^ NaOH (Chem-Supply, Australia). Each solution was neutralized with 1.0 mol L^−1^ H_2_SO_4_ (98%, RCI Labscan, Thailand) and diluted to 100 mL with deionized water. Working standard solutions of As(III) and As(V) were prepared daily by stepwise dilution to the desired concentrations using deionized water. A solution of 0.45 mol L^−1^ HCl was prepared by dilution of concentrated HCl (32%, Ajax, Australia) with deionized water, and 0.3 mol L^−1^ H_2_SO_4_ was prepared by dilution of concentrated H_2_SO_4_ with deionized water. A 1.0% (w/v) sodium borohydride solution (Sigma-Aldrich, Australia) was prepared fresh daily by dissolving 0.10 g of NaBH_4_ in 10 mL of 0.1% (w/v) NaOH solution. Ni(NO_3_)_2_.6H_2_O, KI, and l-ascorbic acid, used in the determination of total As and its speciation, were purchased from Chem-Supply (Australia).

### Design and fabrication of the µPAD

**Fig. 1:**
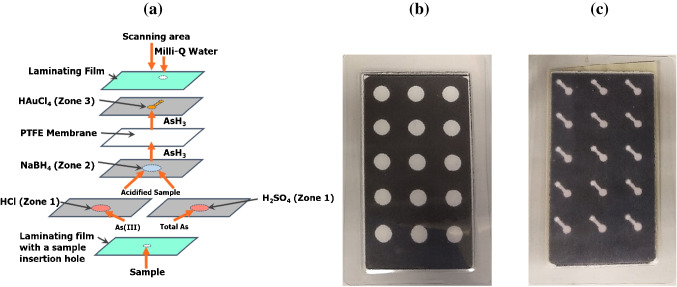
(**a**) Schematic representation of the hydride generation µPAD for inorganic As speciation (only a single sensor is shown). The diameters of zones 1, 2, and 3 are 8 mm, 8 mm, and 3 mm, respectively. Photographic images of the sample zones (**b**) and the detection zones (**c**) of the µPAD

Each μPAD contained 15 sensors and was credit card size (78 mm × 58 mm). It was composed of three layers of patterned paper, each consisting of 15 hydrophilic reagent zones each, and one layer of PTFE tape (Fig. 1).

Details about the fabrication of the μPAD are provided in the Electronic Supplementary Material.

### Analytical procedure

Prior to sample introduction, 1.5 μL of deionized water was deposited through the corresponding insertion hole at the end of the transport channel of each detection zone of the μPAD (Fig. 1c) to rehydrate it. The analytical procedure was conducted at room temperature and involved the deposition of 12 μL of sample or standard solution into each individual sample insertion hole of the laminated μPAD. These holes were subsequently covered with a masking tape to prevent sample evaporation or loss of arsine gas. The sample rehydrated both zones 1 and 2 of each sensor, thus resulting in acidification of NaBH_4_ and the generation of arsine gas which diffused across the PTFE tape and reduced Au(III), present as HAuCl_4_, in the corresponding detection zones (zone 3) to Au nanoparticles. The detection zone side of the μPAD was scanned after 5 min using a conventional flatbed scanner (Cannon LiDE 120, Japan). Scanned images of the μPADs were stored in JPEG format at 1200 dpi. The color intensity of the individual μPAD detection zones was measured using ImageJ software (National Institute of Health USA, http://imagej.nih.gov/ij). The mean red, green, and blue color intensities were obtained from the center of each detection zone. The highest intensity was that of the green color and it was used in the subsequent measurements. The measured color intensity was converted to reflectance by the method of Birch and Stickle [24], i.e., Reflectance = − log(*I*/*I*_0_), where *I* is the mean green color intensity for the sample or standard and *I*_0_ is the mean green color intensity for the blank. Deionized water was used as the blank. As with previous studies [25], outlier reflectance values for each sample or standard were removed by excluding values greater than the 90^th^ or lower than the 10^th^ percentiles of the ranked reflectance values.

### Optimization of the µPAD

Table S1 (Electronic Supplementary Material) presents a list of the main design and operational μPAD parameters which were optimized in the present study together with the investigated ranges, initial values, and optimal values in the order in which the optimization was conducted.

### Stability of the µPAD

The stability of the optimized μPAD with zone 1 (Fig. 1a) impregnated with either HCl or H_2_SO_4_ was studied under two different conditions: (1) at room temperature; and (2) at ≤  − 20 °C (in a freezer). Under both conditions, μPADs were vacuum sealed in FoodSaver Vacuum sealing bags containing a silica desiccant pouch to minimize exposure to moisture. Additionally, under both storage conditions, the µPADs were stored in the dark because of the light sensitivity of HAuCl_4_. The stability of μPADs was assessed by performing daily measurements of the reflectance of their detection zones (zone 3) after the addition of 12 μL of a 5 mg L^−1^ As(III) standard solution. These daily measurements continued until the obtained mean concentration values (*n* = 11) decreased by more than 2 σ_n−1_ from the value obtained with a freshly prepared μPAD.

### Validation

To determine the applicability of the newly developed µPAD method for environmental analysis, it was used in the analysis of groundwater and freshwater samples collected from gold mining regions in the State of Victoria (Australia). The results obtained were validated against those obtained by appropriate reference methods outlined in this section.

For As speciation, As(III) was determined by inductively coupled plasma-optical emission spectrometry (ICP-OES, Optima 4300 DV, Perkin-Elmer) in conjunction with hydride generation (HG). Hydride generation was executed in a 1 M citrate buffer at pH 4.5 in a homemade HG system. Total inorganic As was determined after pre-reducing As(V) to As(III) with KI and ascorbic acid (AA) in 0.4 M HCl solution [26]. As(III) concentration in all samples was found to be significantly lower than the limit of detection (LOD) of the proposed µPAD. Therefore, these samples were spiked with As(III) prior to their µPAD-based As speciation. In order to further validate the newly developed µPAD with different types of sample matrices, water samples from the Ciliwung River in Jakarta (Indonesia) and Indian groundwater were spiked with both As(III) and As(V) prior to analysis.

## Results and discussion

### Selection of the colorimetric reagent

The suitability of the following colorimetric reagents for the detection of As was considered: potassium permanganate [10], sulfanilic acid with N-(1-naphthyl)ethylenediamine dihydrochloride (NEDA) [27], sodium dodecyl sulfate (SDS) micelle bound methylene blue dye with a silver nanoparticle catalyst [28], and gold(III) chloride [21]. The corresponding colorimetric detection methods, except for the one using gold(III) chloride, were discounted due to toxicity, impracticality, and incompatibility with the newly developed µPAD. Gold(III) chloride was the only reagent tested that proved to be both stable on paper and capable of producing adequate sensitivity. Therefore, the corresponding color reaction was chosen for all subsequent experiments.

### Optimization results

According to that reported in the literature methods for the colorimetric determination of arsenic by hydride generation, alkaline sodium borohydride solutions should be prepared daily [29]. Under these conditions, it was investigated whether this reagent could be dried on the paper substrate prior to device lamination and still retain its properties after subsequent rehydration by an acidified sample. The use of solid reagents presents an attractive alternative to that of wet reagents, as this improves the user-friendliness, portability, and utility of the µPADs [19]. The color development of the µPAD using rehydrated by the sample sodium borohydride in preliminary experiments indicated that such an approach was feasible.

The configuration of the μPAD was based on the gas-diffusion μPAD for the determination of ammonia, developed earlier by us [20]. On this basis, the diameter of the detection zone (zone 3) was selected as equal to 3 mm. To ensure complete exposure of the sample to the reducing reagent (i.e., sodium borohydride), it was decided to keep the diameters of the sample zone (zone 1) and reagent zone (zone 2) equal. This diameter determined the sample volume. The effect of sample volume on the reflectance value was studied in the range from 8 to 16 μL by varying the sample zone diameter and best results in terms of reflectance value and precision were obtained for 12 μL corresponding to a diameter of 8 mm for both zone 1 and zone 2 (Electronic Supplementary Material, Fig. S1).

To confirm that the concentration of sodium borohydride recommended in the literature was the optimal concentration when applied to a paper substrate, solutions with different sodium borohydride concentrations within the range 0.050 to 1.5% (w/v) were deposited into zone 2 of the µPAD. The reflectance was found to increase to a maximum value corresponding to a sodium borohydride concentration of 1.0% (w/v), above which it began to decrease. This effect was most likely due to the vigorous generation of hydrogen, which increased the reflectance of the blank, as had been observed in a flow injection method employing the same chemistry [10]. A sodium borohydride concentration of 1.0% (w/v) corresponded to the highest reflectance value. Moreover, this was the concentration used in the flow injection methods mentioned above; and therefore, it was selected for all subsequent experiments.

Solutions of sodium borohydride should be alkaline to ensure the stability of the reagent [30]. This is most often achieved by the addition of sodium hydroxide to reach a solution concentration of 0.1% (w/v), which was used in the present study. Sodium hydroxide was not expected to affect the analytical signal, although a screening test was performed to assess its influence on the reflectance of the µPAD detection zone. The results showed that this influence was insignificant.

The effect of the concentration of gold(III) chloride on the reflectance was studied in the range from 1.0 to 15 mmol L^−1^, and 5 mmol L^−1^ was chosen as the optimum concentration as it provided an acceptable compromise between reflectance and cost of analysis (Electronic Supplementary Material, Fig. S2).

To further improve the utility of the device in the field, it was investigated whether the colorimetric reagent could be dried prior to lamination, and then rehydrated with deionized water before use. This would avoid the necessity to use a separate gold(III) chloride solution in the field measurements. Moreover, deionized water presents a safer and more practical option. It was found that drying and rehydrating the colorimetric reagent increased the sensitivity of the device by about 20% compared to directly introducing the colorimetric reagent solution instead. This may be due to the deposition of the colorimetric reagent directly onto the detection zone prior to drying, rather than via the hydrophilic side channel (Fig. 1). Hence, the approach based on rehydrating the detection zone, impregnated with gold(III) chloride solution and then dried, was used in the subsequent experiments.

A practical consideration for any analytical device intended for field analysis is the time required to conduct a measurement. The time per analysis for commercially available arsenic test kits ranges from 5 to 30 min [14]. The interval required by these systems is determined by the analytical procedure, the sample/reagent volumes used, and the efficiency of mixing. The newly developed µPAD was intended as an alternative to these commercial kits; hence, it was considered important to ascertain if the optimized configuration would allow faster sample analysis. The color development time after the introduction of 12 μL of 5 mg L^−1^ As(III) standard was varied from 1 to 30 min after which imagining was performed. It was found that after an initial increase, the reflectance levelled off between 3 and 7 min, and started to decrease for color development times greater than 7 min, which could be due to slow agglomeration of the gold nanoparticles (zone 3, Fig. 1a). Consequently, 5 min were selected as the optimum color development time.

**Fig. 2:**
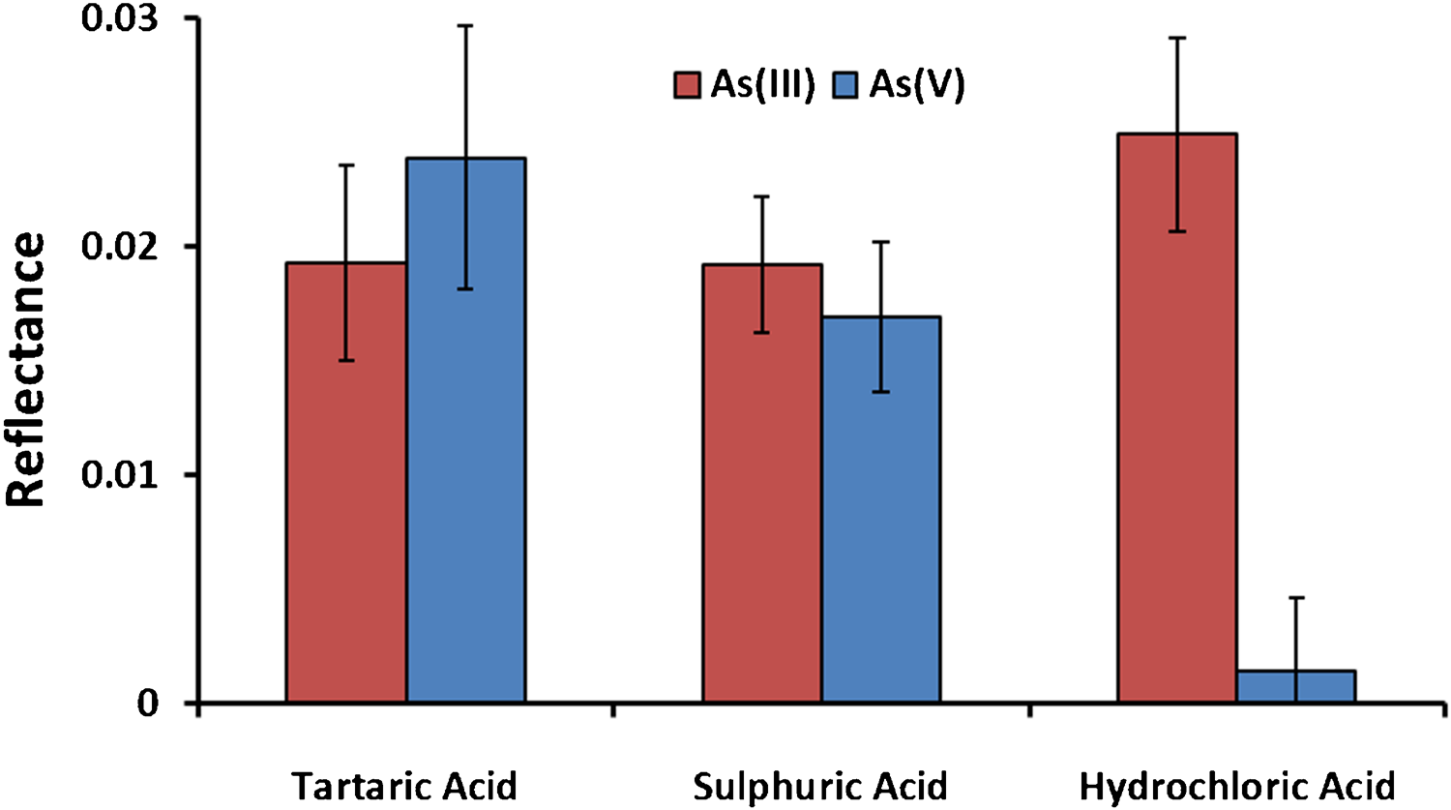
Reflectance values for 12 μL of 5 mg L^−1^ As(III) or 5 mg L^−1^ As(V) in the cases of zone 1 being impregnated with 0.6 M tartaric, 0.3 M sulfuric, or 0.45 M hydrochloric acid and dried afterwards. Remaining experimental conditions: zone 2 impregnated with 12 μL solution containing 1.0% (w/v) NaBH_4_ and 0.1% (w/v) NaOH; zone 3 impregnated with 1.5 μL 5 mM HAuCl_4_ solution; and 5-min color development time. The error bars are ± 1σ_*n*−1_ (*n* = 35)

Numerous hydride generation systems for As speciation have utilized the pH dependence of the reduction of As(III) and As(V) to arsine to allow the determination of the concentration of As(III) and the combined concentration of As(III) and As(V) (i.e., total inorganic As). In this context, nitric, sulfuric, and hydrochloric acids have been commonly employed to acidify As samples in As speciation studies [10–12, 29]. Additionally, tartaric acid has been proposed as a solid reagent for hydride generation analysis [31]. An important step in the development of the µPAD was the determination of the suitability of the acids mentioned above to be dried on paper and subsequently rehydrated by the sample. In the corresponding experiments, zone 1 of the µPAD was impregnated with sulfuric, hydrochloric, or tartaric acid. Earlier experimentation indicated that nitric acid was unsuitable for drying on filter paper due to its oxidizing properties. Of the three acids tested, sulfuric acid was found to provide the greatest equivalency between the As(III) and As(V) reflectance values, in addition to an increased repeatability relative to tartaric acid (Fig. 2). Unexpectedly, when zone 1 was acidified with hydrochloric acid, almost no signal was obtained for As(V) (Fig. 2). It was hypothesized that in the drying process a portion of the relatively volatile hydrochloric acid evaporated, resulting in insufficient acidity for the efficient reduction of As(V) to arsine [32]. To test this hypothesis, instead of drying the hydrochloric acid in zone 1, the As(III)/As(V) standards were acidified using this acid prior to their introduction into the µPAD with its zone 1 left untreated. Under these conditions, the reflectance values for As(III) and As(V) were found to be statistically indistinguishable, as confirmed by the Student *t*-test at a 95% confidence level.

The reflectance values for As(III) and As(V) in the case of tartaric acid were characterized by high variability, and in addition they differed significantly while at the same time they did not allow differentiation between the two As species (Fig. 2). Therefore, tartaric acid was not used further.

Additional experiments were performed to determine the extent of hydrochloric and sulfuric acid loss during the drying process. This was achieved by measuring the equilibrium pH of 25 mL of mechanically stirred deionized water after the immersion of a filter paper disk cut with the same diameter (i.e., 8 mm) as zone 1 (Fig. 1a) onto which 12 μL of acid (i.e., 0.45 M HCl or 0.3 M H_2_SO_4_) had been dried. Experiments were also performed in which 12 μL of the acids mentioned above were added directly to 25 mL of deionized water. The pH data were subsequently used to calculate the theoretical pH of zone 1 of the µPAD in the cases when either dried acid or freshly added acid was used. The results, presented in Fig. S3 (Electronic Supplementary Material), confirmed the hypothesis that a significant fraction of HCl evaporated during the drying of the sample zone while the acidity of this zone was unaffected when sulfuric acid was used instead.

This difference in the acidity of zone 1, which was in agreement with the results presented in Fig. 2, indicated the possibility of using µPADs with their zone 1 impregnated with either H_2_SO_4_ or HCl solutions for inorganic As speciation. Therefore, the influence of the concentrations of these acids on the efficiency of inorganic As speciation was studied.

**Fig. 3:**
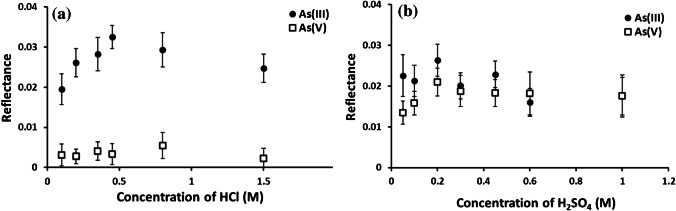
Effect of the HCl (**a**) or H_2_SO_4_ (**b**) concentration in 12 μL solution deposited into zone 1 and dried afterwards on the reflectance of 12 μL standards containing either 5 mg L^−1^ As(III) (black circle) or 5 mg L^−1^ As(V) (white square). Remaining experimental conditions as in Fig. S1 (Electronic Supplementary Material). The error bars are ± 1σ_*n*−1_ (*n* = 35)

The effect of the HCl and H_2_SO_4_ concentrations on the reflectance values for 5 mg L^−1^ As(III) and As(V) is illustrated in Fig. 3. In these experiments, 12 μL of HCl or H_2_SO_4_ solutions with concentrations in the range 0.10–1.5 mol L^−1^ or 0.05–1.0 mol L^−1^, respectively, were deposited into zone 1 and subsequently dried. In the case of HCl, the difference between the corresponding reflectance values increased with increasing the acid concentration and reached a maximum at 0.45 mol L^−1^ (Fig. 3a). A slight decrease was observed at higher concentrations which was attributed to the increase in hydrogen generation at higher acidity leading to higher blank reflectance values. Therefore, 0.45 mol L^−1^ was selected as the optimal HCl concentration. This concentration provided not only the highest As(III) reflectance but also the reflectance for As(V) was negligible, thus allowing the determination of As(III) in the presence of As(V). In the case of H_2_SO_4_, the reflectance for As(III) was found to be relatively independent of the acid concentration, while that for As(V) initially increased with the acid concentration and then levelled off at 0.2 mol L^−1^ (Fig. 3b). For concentrations equal or higher than 0.3 mol L^−1^, the reflectance values for As(III) and As(V) were statistically indistinguishable according to the two-sided *t*-test. On the basis of these results (Fig. 3b), 0.3 mol L^−1^ was selected as the optimal H_2_SO_4_ concentration which would allow the determination of total inorganic As.

### Analytical figures of merit

The analytical performance of the μPAD for the determination of As(III) and total inorganic As was evaluated under optimal conditions (Electronic Supplementary Material, Table S1) and the corresponding analytical figures of merit are summarized in Table 1. The LOD was determined by the linear regression method of Miller and Miller [33]. These results indicate that the newly developed μPAD is suitable for the speciation of inorganic arsenic in irrigation waters as the LOD values are lower than the Australian and New Zealand guideline value of 2 mg L^−1^ [4].

**Table 1:** Analytical figures of merit of the newly developed μPAD-based method for the determination of total inorganic As (T-As) and As(III) under optimal conditions (Electronic Supplementary Material, Table S1)

Parameter	T-As	As(III)
Linear range (mg L^−1^)	1.2– 8.0	1.2 – 8.0
Limit of detection (mg L^−1^)	0.43	0.41
Intra-device repeatability expressed as RSD (%) for 3 µPADs	8.22, 7.68, 4.78	6.03, 4.16, 7.68
Inter-device repeatability expressed as RSD (%) for 3 µPADs	6.91	6.74

The calibration curves including the standard errors of the regression coefficients for T-As and As(III) determination are described by Eqs. (1) and (2), respectively.

1 $${R}_{T-As}=\left(3.50\times {10}^{-3}\pm 0.31\times {10}^{-3}\right)\times {C}_{T-As}+\left(3.09\times {10}^{-3}\pm 6.35\times {10}^{-5}\right), {(R}^{2}=0.998)$$

2 $${R}_{As(III)}=\left(4.71\times {10}^{-3}\pm 0.21\times {10}^{-3}\right)\times {C}_{As\left(III\right)}+\left(2.53\times {10}^{-3}\pm 1.06\times {10}^{-3}\right), ({R}^{2}=0.992)$$

where *R* is reflectance, *C* is concentration (expressed in mg As L^−1^), and subscripts T-As and As(III) refer to total inorganic As and As(III), respectively.

The precision of the μPAD-based method was characterized by the inter- and intra-µPAD repeatability (Table 1). The intra-µPAD repeatability was assessed by measuring the reflectance for 6.5 mg L^−1^ As(III) standards on 3 µPADs and was expressed as the relative standard deviation (RSD) of the individual devices (*n* = 11). The inter-µPAD repeatability was obtained as the RSD of the average reflectance values of the same 3 µPADs (*n* = 35). The repeatability data presented in Table 1 indicate that the reliability of the µPAD-based method exceeds that of the methods utilizing commercial arsenic test kits, which are usually used as screening tools [14]. Additionally, the precision of the newly developed µPAD-based method is consistent with that of other µPAD-based methods described in the literature [19, 20].

The limit of detection of the newly developed μPAD-based method (Table 1) for the determination of both As(III) and T-As is higher compared to those reported for previously developed μPADs (i.e., > 10 μg L^−1^ [21], 1 μg L^−1^ [22], and 1 mg L^−1^ [23]). This difference is the result of the gas-diffusion separation step which allowed the newly developed μPAD to be used for inorganic As speciation unlike the other μPADs mentioned above which could be used for As(III) determination only. Due to its relatively high limit of detection, the newly developed μPAD is not suitable for As speciation in drinking water or less polluted environmental waters.

The detection reaction in the newly developed μPAD is based on arsine reducing HAuCl_4_ in the device’s detection zone. The detection of As(III) in the previously developed μPADs involves the use of Au nanoparticles prepared through complex functionalization procedures (i.e., step-wise conjugation with thioctic acid and thioguanine [21, 22] or functionalization with negatively charged DNA aptamers [23]). Therefore, it can be expected that the production costs of the newly developed μPAD will be lower than those for the other μPADs, mentioned above.

### Interference and stability studies

The potential interference of ions, commonly found in environmental waters, on the µPAD-based analysis of 5 mg L^−1^ As(III) or 5 mg L^−1^ As(V) standards was studied. Lack of interference was assumed when the deviation from 100% recovery was not greater than 5%. Table 2 lists the tolerable concentrations of the potential interferents studied and their short-term trigger values [4].

**Table 2:** Tolerable concentrations of ions commonly present in environmental waters at which they did not interfere with the determination of 5 mg L^−1^ As(III) or As(V) standard by the newly developed µPAD by more than 5% (*n* = 35)

Interferent	Short-term irrigation trigger value (mg L^−1^) [4]	Tolerable concentration (mg L^−1^)
Lead(II)	5	100
Cobalt(II)	0.1	50
Copper(II)	5	50
Iron(II/III)	10	30
Nickel(II)	2	50
Selenium(IV)	0.05	5
Antimony(III)	N/A	5
Bismuth(III)	N/A	50
Magnesium(II)	N/A	100
Calcium(II)	N/A	500
Phosphate	0.8—12	500
Sulfate	N/A	500
Nitrate	N/A	500
Chloride	N/A	500
Carbonate	N/A	500

*N/A*, not applicable.

Table 2 shows that the newly developed µPAD-based method can tolerate the common hydride-forming ions such as Se(IV) and Sb(III) up to an equivalent concentration of As(III)/As(V). This is to be expected as these cations can form volatile hydrides that can react with gold(III) chloride in zone 3 (Fig. 1a). It should be noted that selenium and antimony are not present in environmental waters at concentrations higher than those of inorganic arsenic species [2]; hence, it is unlikely that such interference would have any practical significance for arsenic analysis and speciation. Bi(III) could be tolerated at concentrations up to 10 times higher than that of arsenic and the tolerance levels for other common transition metal cations are greater than their respective short-term irrigation trigger values. Additionally, common anionic species were found to be tolerated at concentrations exceeding 100 times that of arsenic.

At room temperature, the stability of the HCl-based μPAD was 2 days while the H_2_SO_4_-based device was stable for 7 days. However, when stored in a freezer, the μPAD was stable for at least 20 days irrespective of the acid used.

### Analysis of environmental water samples

A selection of groundwater and freshwater samples (samples 1 to 3, Table 3) from Victorian gold mining regions were analyzed using the newly developed µPAD. The concentration of As(III) in all samples was found to be significantly lower than the LOD of the µPAD-based method. Therefore, the samples were spiked with As(III) at different levels. In order to further examine the applicability of the µPADs for the analysis of samples with different types of matrices, samples of water from the Ciliwung River (Sample 4) in Jakarta and Indian groundwater (Sample 5) were spiked with As(III) and As(V) prior to analysis. For all samples, the spiked concentrations were regarded as the true arsenic concentration values. Table 3 illustrates the good recovery percentages for As(III) for all 5 samples mentioned above obtained by the newly developed µPAD. The same table also shows the good agreement between the total inorganic arsenic concentration values determined by the μPAD-based method and the total inorganic arsenic concentration values calculated as the sum of the spiked As(III) concentrations and the As(V) concentrations either spiked (samples 4 and 5) or determined by HG-ICP-OES (samples 1–3). The total inorganic arsenic concentration values, determined by the μPAD-based method, were found by Student’s *t*-test at the 95% confidence level to be statistically indistinguishable from those calculated.

**Table 3:** Determination of As(III) and total inorganic arsenic (T-As) in environmental water samples by the newly developed µPAD (*n* = 35 per sample). Results are presented as average ± 95% confidence interval

Sample	As(III)			As(V)	T-As	
	Spiked (mg L^−1^)	µPAD (mg L^−1^)	Recovery^*^ (%)	HG-ICP-OES/spiked (mg L^−1^)	Calculated^&^ (mg L^−1^)	µPAD (mg L^−1^)
1	1.50	1.60 ± 0.19	106	1.38 ± 0.09^#^	2.88 ± 0.09	2.70 ± 0.25
2	1.00	1.18 ± 0.22	118	1.87 ± 0.03^#^	2.87 ± 0.03	2.71 ± 0.16
3	2.00	1.92 ± 0.23	95.9	0.99 ± 0.07^#^	2.99 ± 0.07	2.73 ± 0.24
4	2.10	2.26 ± 0.18	108	2.25^##^	4.35	4.65 ± 0.32
5	1.95	1.90 ± 0.10	97.3	3.05^##^	5.00	4.83 ± 0.28

*As(III) Recovery (%) = As(III)_µPAD_ / As(III)_spiked_ × 100.

^#^Determined by HG-ICP-OES (*n* = 3 per sample).

^##^Spiked.

^&^T-As_Calculated_ = As(V)_HG-ICP-OES/Spiked_ + As(III)_Spiked_.

The results shown in Table 3 demonstrate the suitability of the µPAD for speciation of inorganic arsenic in environmental aqueous samples.

## Conclusions

The newly developed µPAD utilized for the first-time hydride generation on a paper-based platform to allow speciation of inorganic As in complex water samples. The sensitivity offered by this device, while not suitable for drinking and pristine environmental waters, is adequate for As analysis of agricultural irrigation waters where the maximum allowed As concentration is 2 mg L^−1^. The µPAD is also a disposable low-cost and user-friendly device (approximately $0.30 per determination) which allows relatively fast inorganic As speciation in waters contaminated with this metalloid, such as agricultural waters and waters from the mining industry (e.g., gold mining) where elevated concentrations of As are not uncommon.

The successful utilization of hydride generation on a paper-based platform opens new horizons in paper-based microfluids with respect to on-line pretreatment of complex samples and will lead to the development of µPADs for other hydride-forming elements.

## Supplementary Information

Below is the link to the electronic supplementary material.

Supplementary file1 (PDF 382 KB)
