# Supplementary material for: Speciation of inorganic arsenic in aqueous samples using a novel hydride generation microfluidic paper-based analytical device (µPAD)
Source: Mikrochim Acta. 2022 Jun 3;189(7):243. doi: 10.1007/s00604-022-05339-w (PMC9166862; doi:10.1007/s00604-022-05339-w)
Supplement: Supplementary file 1 — Supplementary file1 (PDF 382 KB) [file 604_2022_5339_MOESM1_ESM.pdf]

Electronic Supplementary Material

Speciation of inorganic arsenic in aqueous samples using a novel hydride generation microfluidic paper-based analytical device (µPAD)

Mason E. Bonacci, M. Inês G. S. Almeida*, Yanlin Zhang, Spas D. Kolev*

School of Chemistry, The University of Melbourne, Victoria 3010, Australia

Design and fabrication of the µPAD

Wax printing (Xerox Colorqube 8870) was used to create hydrophilic zones by patterning the filter paper (Grade 4, Whatman) with hydrophobic wax. The templates were created in Adobe Photoshop. The wax-printed filter paper was placed in an oven (Binder, USA) at 150 °C for 120 s to allow the wax to melt and penetrate the cellulose fibers.

The fabrication of a 3D µPAD involved the deposition of 12 µL of acid in each sample zone (Zone 1, Fig. 1a). Hydrochloric acid (0.45 mol L^{-1}) or sulfuric acid (0.30 mol L^{-1}) were used for the determination of As(III) or total inorganic As, respectively. The reagent zone (Zone 2, Fig. 1a) and the detection zone linked to a short (2 mm) transport channel (Zone 3, Fig. 1a) were impregnated with 12 µL alkaline sodium borohydride solution (1% (w/v) $NaBH_4$ and 0.1% (w/v) NaOH) and 1.5 µL of 5 mM $HAuCl_4$ solution, respectively. After the addition of the reagent solutions, mentioned above, to their respective zones, the µPAD was oven-dried for 5 min at 40 °C. The paper layer with the sodium borohydride reagent zones (Zone 2, Fig. 1a) was separated from the paper layer with the detection zones (Zone 3) by 3 parallel strips of PTFE hydrophobic microporous tape (Bridgland, Australia; 12 mm × 84 mm × 0.075 mm thickness). After ensuring that all 3 zones of the individual sensors were well aligned, the µPAD was laminated (GBC Triad A4) to maintain this alignment and prevent the evaporation of the sample and $HAuCl_4$ solutions during the detection process [1]. A Japanese screw punch was used to punch a sample insertion hole of 2 mm in diameter in the plastic cover over the center of each sample zone (Zone 1, Fig. 1a), and another insertion hole at the end of the transport channel of each detection zone (Zone 3) used for introducing deionized water.

*Corresponding Authors. Emails: s.kolev@unimelb.edu.au (S.D. Kolev), ines.almeida@unimelb.edu.au (M.I.G.S. Almeida)

Table S1. Summary of the µPAD parameters optimized in the present study.

Parameter	Range studied	Optimal value	Initial value
Sample volume (µL)	8 – 16	12	N/A
Zone 2: $NaBH_4$ concentration (%(w/v))	0.050 –1.5	1.0	1.0
Zone 3: $HAuCl_4$ concentration (mmol L^{-1})	1.0 – 15	5.0	5.0
Color development time (min)	1 – 30	5	10
Zone 1: HCl concentration (mol L^{-1})	0.10 – 1.5	0.45	0.8
Zone 1: H_2SO_4 concentration (mol L^{-1})	0.10 – 1.0	0.30	N/A

Influence of the sample volume

As expected, the reflectance value increased with the initial increase of the sample volume up to 12 µL and thereafter remained practically unchanged (Fig. S1). While a sample volume of 14 µL provided the highest reflectance value, a 12 µL sample volume was chosen for all subsequent experiments as it provided significantly greater precision. Therefore, a diameter of 8 mm for both Zone 1 and Zone 2 (Fig. 1) was used in all subsequent experiments.

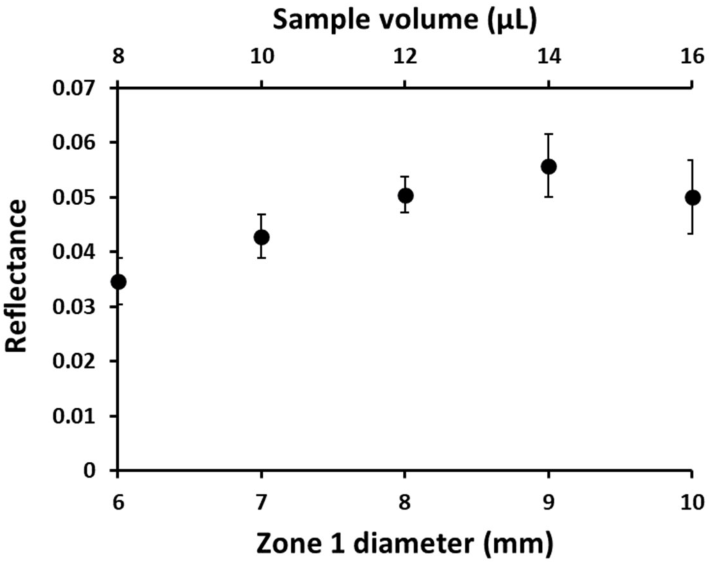

Figure S1. Effect of the sample volume/Zone 1 diameter (Fig. 1) on the reflectance of 12 µL of 5 mg L^{-1} As(III) standard. Zone 1 was impregnated with 0.8 M HCl solution and dried afterwards. Zone 2 was impregnated with a solution containing 1.0% (w/v) $NaBH_4$ and 0.1% (w/v) NaOH. The color development time was 10 min. The error bars are ± $1\sigma_{n-1}$ (n = 35).

Influence of the gold(III) chloride concentration

It was observed that the reflectance increased initially with increasing the gold(III) chloride

($HAuCl_4$) concentration and levelled off at 2.5 mmol L^{-1} (Fig. S2). There was a slight increase in the reflectance for higher concentrations of gold(III) chloride, however, because of the high price of this reagent, 5 mmol L^{-1} was chosen as the optimum concentration as it provided an acceptable compromise between reflectance and cost of analysis.

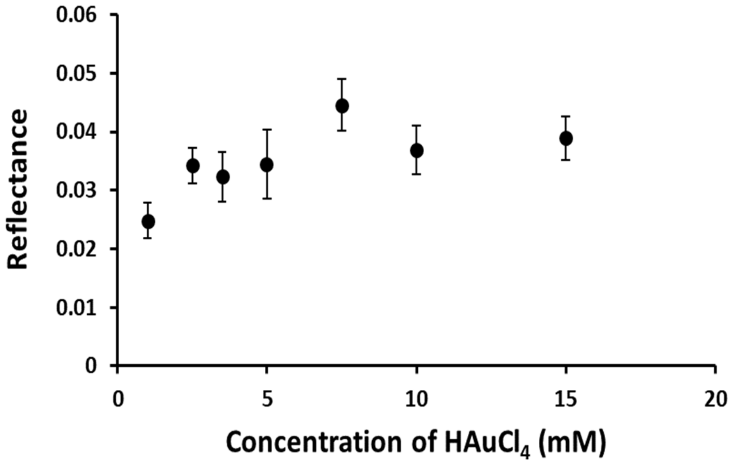

Figure S2. Effect of the gold(III) chloride concentration in 1.5 μL solution deposited into Zone 3 (Fig. 1) on the reflectance of 12 μL of 5 mg L^{-1} As(III) standard. Zone 1 was impregnated with 0.8 M HCl solution and dried afterwards while Zone 2 was impregnated with a 12 μL solution containing 1.0% (w/v) $NaBH_4$ and 0.1% (w/v) NaOH. The color development time was 10 min. The error bars are ± $1\sigma_{n-1}$ (n = 35).

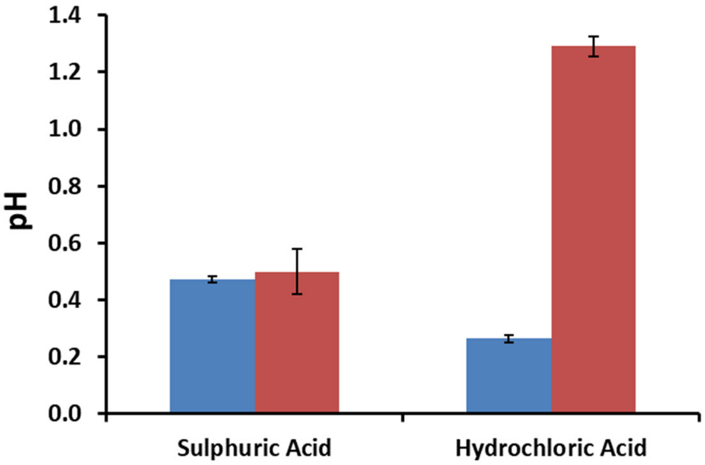

Figure S3. pH of Zone 1 (Fig. 1) in the cases of using dried (■) or freshly added (■) 12 μL of 0.3 M H_2SO_4 or 0.45 M HCl solutions. The error bars are ± $1\sigma_{n-1}$ (n = 6).

References

1. Jayawardane BM, McKelvie ID, Kolev SD (2015) Development of a gas-diffusion microfluidic paper-based analytical device (μPAD) for the determination of ammonia in wastewater samples. Anal Chem 87 (9):4621-4626. doi:https://doi.org/10.1021/acs.analchem.5b00125
